# Population Genomics of North Carolina’s Black Bears (*Ursus americanus*) Reveals Substructure and Identifies Informative SNPs for Population Assignment and Individualization

**DOI:** 10.3390/genes17101206

**Published:** 2026-09-29

**Authors:** Isabella G. Livingston, Allison N. Dickey, Megan N. Dillon, Nicholas P. Gould, Jennifer Strules, Christopher S. DePerno, Michael K. Stoskopf, Kelly A. Meiklejohn, Matthew Breen

**Affiliations:** 1 Department of Molecular Biomedical Sciences, College of Veterinary Medicine, North Carolina State University, Raleigh, NC 27607, USA; igliving@ncsu.edu (I.G.L.); mdillon3@ncsu.edu (M.N.D.); 2 Bioinformatics Research Center, North Carolina State University, Raleigh, NC 27607, USA; andickey@ncsu.edu; 3 College of Natural Resources, North Carolina State University, Raleigh, NC 27695, USA; ngould7@msn.com (N.P.G.); jstrules@gmail.com (J.S.); csdepern@ncsu.edu (C.S.D.); 4 Department of Clinical Sciences, College of Veterinary Medicine, North Carolina State University, Raleigh, NC 27607, USA; mks@stoskopf.net; 5 Department of Population Health and Pathobiology, College of Veterinary Medicine, North Carolina State University, Raleigh, NC 27607, USA; kameikle@ncsu.edu; 6 School of Science, Hawkesbury, Western Sydney University, Richmond, NSW 2753, Australia; 7 Center for Human Health and the Environment, North Carolina State University, Raleigh, NC 27607, USA; 8 Comparative Medicine Institute, North Carolina State University, Raleigh, NC 27607, USA; 9 Cancer Genetics, UNC Lineberger Comprehensive Cancer Center, University of North Carolina, Chapel Hill, NC 27599, USA; 10 Duke Cancer Institute, Duke University, Durham, NC 27710, USA

**Keywords:** conservation genomics, genetic diversity, *Ursus americanus*, population substructure, animals, wildlife management

## Abstract

**Background/Objectives**: Anthropogenic pressures increasingly fragment populations, reduce connectivity in large mammals, and threaten genetic diversity. Genetic diversity is essential for a population’s ability to respond to environmental change and the long-term persistence of any species. The American black bear (*Ursus americanus*) is an important ecological regulator of many ecosystems. North Carolina (NC) is home to a large number of black bears that contribute to ecosystem function. In this study, we examined how rapidly changing environmental and anthropogenic effects influence the structure and genetic health of black bears in North Carolina. **Methods**: Whole genome sequencing data were generated from 82 black bears selected from populations representing two of the three state recognized management units, the Mountain Bear Management Unit (MBMU, *n* = 49) and the Coastal Bear Management Unit (CBMU, *n* = 27). Of the 82 black bears, 76 were retained after quality and relatedness filtering. These whole genome sequence data were used to identify almost 900,000 single nucleotide polymorphisms (SNPs) for population genetic analyses. **Results**: The data identified three genetic clusters, one within the CBMU and two within the MBMU, indicating regional substructure across the sampled populations. Compared with the MBMU, the CBMU exhibited significantly lower genetic diversity and higher inbreeding (*p* < 2.2 × 10^−16^), suggesting reduced connectivity and increased genetic vulnerability in the coastal population. Finally, a candidate panel of 4990 SNPs was developed that can be used for the population and individual assignment of black bears. **Conclusions**: These findings provide a valuable genome-wide resource for black bear conservation in North Carolina and support management strategies aimed at maintaining connectivity, preserving genetic diversity, and promoting long-term population resilience.

## 1. Introduction

The American black bear (*Ursus americanus*), an opportunistic omnivore occupying high trophic levels, serves critical ecological roles as a seed disperser, ecosystem regulator, and an indicator of forest health across North America [1,2,3]. Historically widespread across North American forests from Alaska to Mexico, black bears now occupy less than 70% of their original range due to unregulated harvest, persecution, and extensive habitat alteration [4,5,6,7,8]. In the southeastern United States, habitat loss contributed to a population reduction to as low as 10% of the historical numbers, fragmenting once contiguous populations into smaller, isolated units [2,4,6,8].

Despite substantial population recovery in recent decades, black bears continue to face pressures from habitat fragmentation, expanding road networks, and urban encroachment that restrict movement and reduce connectivity [6]. These anthropogenic pressures can erode genetic diversity and adaptive potential, thereby increasing the extinction risk, especially for large mammals with big home ranges and slow reproductive rates [4,8,9,10,11]. Black bears exhibit high behavioral plasticity and can persist in urban landscapes by exploiting near year-round anthropogenic food sources and adjusting activity patterns to be more crepuscular/nocturnal to avoid human activity [7,8,12,13,14,15]. However, over 70% of subadult and adult black bear mortalities result from human activities, including legal harvest, vehicle collisions, gunshot wounds, and conflict-related management actions [13,15,16]. While adaptability has enabled population recovery, it has also increased human–bear conflict [7,17,18], creating management challenges that require balancing conservation goals with human safety and tolerance.

In North Carolina (NC), black bears were nearly extirpated by the mid-20th century, with fewer than 1000 individuals confined to remote mountain and coastal landscapes [13,19]. Following the establishment of the Bear Sanctuary System and the North Carolina Wildlife Resources Commission (NCWRC) Bear Management Plan in 1981, which emphasized regulated harvest, habitat conservation, and the creation of Bear Management Areas, the population rebounded dramatically to an estimated >20,000 individuals by 2022 [19]. These bears are divided into three management units (MUs): the Mountain Bear Management Unit (MBMU, ~8000 bears), the Piedmont Bear Management Unit (PBMU, <1000 bears), and the Coastal Bear Management Unit (CBMU, ~11,000 bears). Although populations have recovered in both the mountain and coastal regions, the central Piedmont region, dominated by dense human populations and extensive transportation infrastructure, is still largely unoccupied, forming less than 5% of the total NC black bear population.

**Figure 1:**
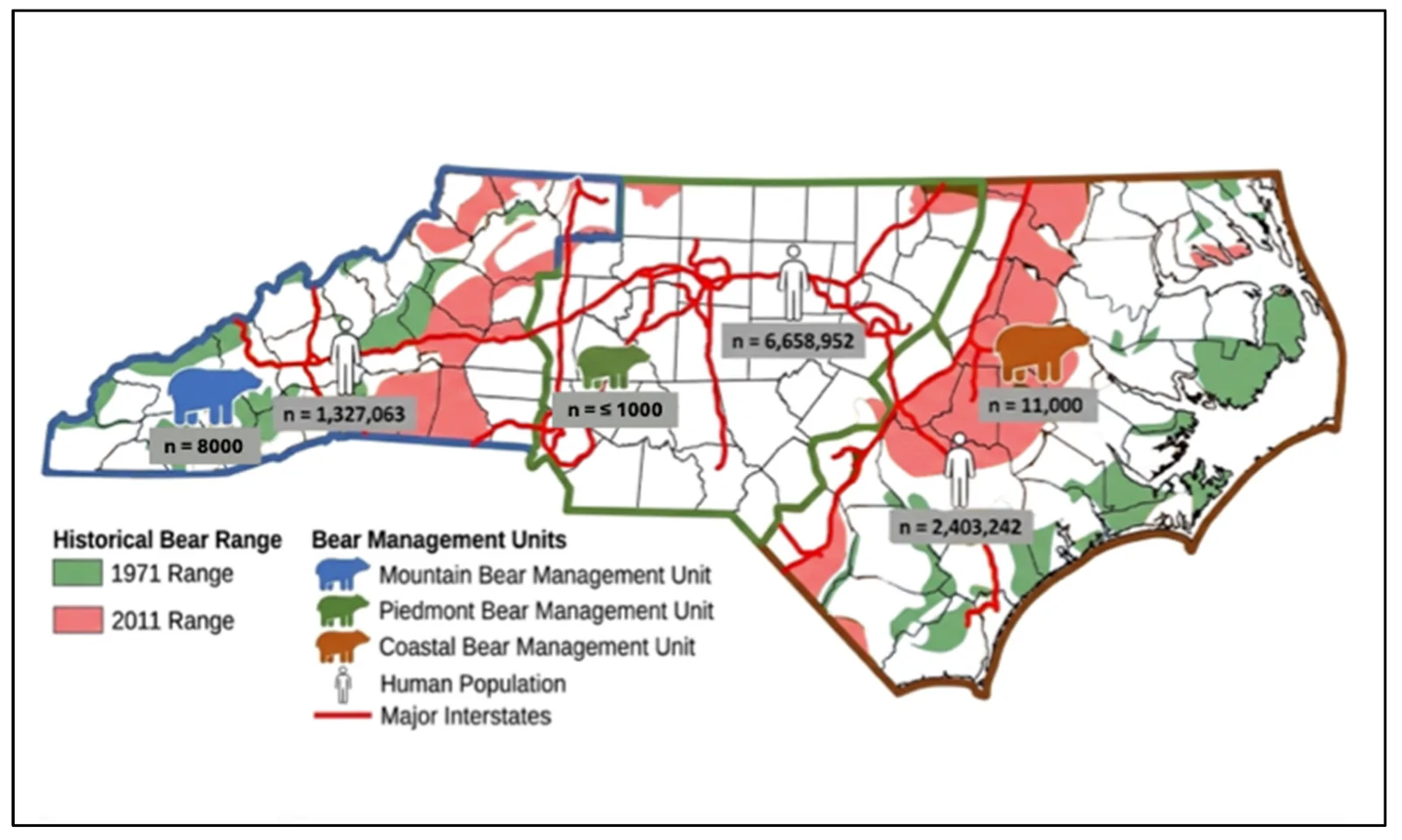
Human populations, estimated black bear populations, historical bear ranges, and bear management unit boundaries in North Carolina. Colored outlines (blue, green, brown) delineate the Mountain, Piedmont, and Coastal Bear Management Units, and red lines represent major interstates. Shaded areas show estimated black bear ranges in 1971 and 2011, illustrating broad range expansion toward the Piedmont region over time. Human population estimates were obtained from the most recent census data available, while bear population estimates and range boundaries were adapted from the North Carolina Wildlife Resources Commission Black Bear Management Plan 2022.

Historical range expansion data suggest that bears are expanding toward the Piedmont but have yet to establish breeding populations in these developed areas, suggesting that this anthropogenic landscape contributes to restricted connectivity between the MBMU and CBMU (Figure 1) [19]. North Carolina is also one of the fastest-growing states in the United States, having increased from roughly 10.4 to 11.2 million residents between 2020 and 2025 (~7% growth) [20,21,22]. This rapid expansion of human populations and associated infrastructure is concentrated in urban and suburban corridors across the Piedmont, reinforcing functional barriers to black bear dispersal between the MBMU and CBMU. Balancing population growth and connectivity with human tolerance remains a key management challenge as both bear and human populations continue to expand.

In response to this population growth, state and federal biologists have emphasized the need for management strategies that safeguard the genetic and demographic integrity of black bear populations while minimizing negative human–bear interactions associated with high population densities and habitat overlap between the two species [3]. Effective management therefore requires tools capable of resolving population structure, connectivity, and individual identity. Population genetic analyses provide a powerful framework for understanding wildlife population structure, connectivity, and demographic history, directly informing management decisions [23,24,25,26,27]. Historically, short tandem repeats (STRs), or microsatellites, have been the predominant genetic marker system for wildlife monitoring, although these are increasingly being complemented or replaced by the analysis of single nucleotide polymorphisms (SNPs) [25,28,29]. While STRs offer cost-effectiveness and extensive historical datasets, they are more prone to genotyping errors (e.g., stutter, allelic dropout, inconsistent allele calling) and can be difficult to standardize across laboratories due to differences in scoring protocols and platforms, limiting the comparability of datasets and constraining their utility for large-scale, multi-agency monitoring [25,30,31,32]. SNPs offer millions of genome-wide variants with greater reproducibility, automation potential, and improved power for detecting fine-scale population structure and individual identity [25,30]. Even a few hundred well-selected SNPs can yield probabilities of identity that are several orders of magnitude lower than those typically achieved with standard STR panels, substantially increasing the precision and reliability of individual identification [33,34,35,36].

Despite these advantages, no comprehensive SNP-based population genomic analysis has been conducted for NC black bears. Previous work using the UrsaPlex microsatellite panel (11 autosomal and three sex-linked markers) [1] revealed strong genetic differentiation between the CBMU and MBMU, with further clustering analyses indicating substructure within the MBMU [37]. However, the limited number of STR loci restricts the resolution of fine-scale structure, genomic diversity, recent migration, and individualization potential, underscoring the need for higher-resolution genomic tools to inform region-specific management strategies.

This study expands the genomic monitoring of American black bears across North Carolina’s two largest management units, which differ substantially in geography, habitat composition, and landscape connectivity. We conducted whole-genome sequencing (WGS) of 82 black bears and used a quality-filtered dataset of 76 individuals to characterize genetic differentiation, population structure, contemporary migration, genomic diversity, and inbreeding within and between the CBMU and MBMU. We also developed a candidate targeted SNP panel for population assignment and individualization. By integrating genome-wide patterns with the geographic and landscape context of each MU, this study establishes a genomic baseline for monitoring connectivity, population stability, and for evaluating the long-term effects of habitat change and fragmentation in North Carolina’s black bear populations.

## 2. Materials and Methods

### 2.1. Sample Collection and DNA Extraction

Eighty-two black bears from across NC were selected for WGS (Table 1). Of these, 74 bears were chosen from those analyzed in Badgett et al. [37], selected based on each bear having high-quality (high molecular weight, non-fragmented) genomic DNA and a complete UrsaPlex STR profile. These 74 bears represented all three primary genetic clusters identified by the UrsaPlex STR panel, with two clusters in the MBMU (51 bears) and one in the CBMU (23 bears). The remaining eight DNA samples were from buccal swabs of CBMU bears, collected by the NCWRC (Supplementary Table S1).

Buccal swabs were processed using the Qiagen DNeasy Blood and Tissue Kit (Qiagen, Hilden, Germany) with the following modifications: each swab was snapped near the tip and placed into a labeled 2 mL tube containing 180 µL of Buffer ATL and 20 µL of Proteinase K. Samples were vortexed, briefly centrifuged, and incubated at 56 °C for 15 min. After incubation and thorough vortexing, 400 µL of a 50:50 mixture of Buffer AL and 100% ethanol was added to each sample. Samples were vortexed and centrifuged (20,800× *g*, 1 min), and the supernatant was transferred to a spin column and centrifuged again (20,800× *g*, 1 min), before discarding the flow-through. Columns were washed sequentially with 500 µL of Buffer AW1 and 500 µL of Buffer AW2, centrifuging (20,800× *g*, 1 min) and discarding the flow-through after each wash. Columns were centrifuged for 3 min (20,800× *g*) to dry the membrane following the addition of Buffer AW2. DNA was eluted in 100 µL of Buffer AE, quantified using a Promega Quantus Fluorometer (Promega, Madison, WI, USA) and assessed by spectrophotometry and agarose gel electrophoresis. All sample extracts were stored at −20 °C prior to future use.

### 2.2. Library Preparation and Whole Genome Sequencing

DNA libraries for all 82 DNA samples were prepared using the Illumina DNA PCR-Free Prep Kit (Illumina, San Diego, CA, USA), which uses enzymatic fragmentation without PCR amplification. For samples with DNA concentrations above 100 ng/µL, the Standard Input Protocol (requiring 500 ng input DNA) was followed (*n* = 66). For samples below 100 ng/µL, the Low Input Protocol (requiring 100 ng input DNA) was followed (*n* = 16). Libraries were quantified and pooled to produce a final library concentration of 2.5 nM (approximately 0.04 nM per library) and then submitted for 150 bp paired-end sequencing across four lanes of an Illumina S4 flow cell on a NovaSeq 6000 instrument (Illumina, San Diego, CA, USA), targeting a read depth of 15×.

### 2.3. Variant Calling and SNP Selection

The sequencing reads were quality trimmed, and adapters were removed using fastp [v 0.21.0] [38]. The trimmed reads were mapped to an American black bear genome assembly (NCBI accession GCF_020975775.1—gsc_jax_bbear_1.0) using BWA-MEM [v 0.7.17] with the -M option and the -R option for assigning read groups [39]. The GCF_020975775.1 assembly includes 2212 contigs and one MT chromosome. The read alignment files were cleaned using the Picard [v 2.25.6] CleanSam tool with the VALIDATION_STRINGENCY option set to LENIENT (http://broadinstitute.github.io/picard/ accessed on 21 September 2026). Samtools [v 1.12] was used to merge the BAM files from the four lanes for each sample and to sort the merged BAM files [40]. Duplicate reads were marked with the Genome Analysis Toolkit (GATK) [v 4.2.0.0] MarkDuplicatesSpark tool [41,42].

Variant calling was performed using the GATK [v 4.2.0.0] HaplotypeCaller, CombineGVCFs, and GenotypeGVCFs tools. The HaplotypeCaller was used to call SNPs and indels in each sample and included the -ERC GVCF option. The HaplotypeCaller output files for the 82 samples were combined using CombineGVCFs, and joint genotyping was performed using GenotypeGVCFs. CombineGVCFs and GenotypeGVCFs were run separately for the assembly contigs using the -L option. The MT chromosome was not included in this step. The Picard [v 2.25.6] MergeVcfs tool was used to combine the 2212 VCF files into a single VCF file that included 16,384,269 SNPs and 4,339,675 indels.

The SNPs and indels were subset from the VCF file and filtered using the GATK [v 4.2.0.0] SelectVariants and VariantFiltration tools. The SNP filters included QD < 2.0, QUAL < 30.0, SOR > 3.0, FS > 60.0, MQ < 40.0, MQRankSum < −12.5, and ReadPosRankSum < −8.0. The indel filters included QD < 2.0, QUAL < 30.0, FS > 200.0, and ReadPosRankSum < −20.0. The SelectVariants tool was used to remove SNPs and indels that did not pass the filters, and the remaining variants included 14,464,574 SNPs and 3,957,549 indels.

Variants for all 82 individuals were filtered using PLINK v2.0 (www.cog-genomics.org/plink/2.0/ accessed on 21 September 2026). Variants with any missing genotype calls were excluded (--geno 0) to retain a complete genotype dataset for downstream analyses, and variants with a minor allele frequency (MAF) < 0.05 were removed (--maf 0.05) to reduce the sensitivity of population-structure analyses to very low-frequency alleles and genotype uncertainty. Individuals with more than 40% missing data were removed prior to variant-level filtering, a threshold selected based on the distribution of sample-level missingness to exclude low-quality outliers prior to variant filtering. To minimize bias from close relatives, one individual from each first-degree relative pair was removed using the KING-robust kinship estimator and a cutoff of 0.177 (--king-cutoff-table 0.177), the conventional boundary between first- and second-degree relationships. To ensure independence among markers, variants were pruned for linkage disequilibrium using a sliding window approach (--indep-pairwise 50 5 0.5). This procedure examines 50-SNP windows, shifting by 5 SNPs to allow overlapping windows, and removes one SNP from any pair with r^2^ > 0.5, iterating until no pairs exceed this threshold. Window size and r^2^ threshold were chosen following standard practices for population structure analyses to balance the retention of informative variation while removing tightly linked variants.

Hardy–Weinberg equilibrium (HWE) was evaluated for all loci using exact tests implemented in PLINK2. Tests were conducted for the full dataset and separately within each management unit. The distribution of HWE *p*-values was used to assess potential contributions of population structure, inbreeding, and technical artifacts. Loci were not excluded from genome-wide analyses based solely on HWE deviation, which is expected under structured populations.

### 2.4. Population Structure and Differentiation

To investigate population structure, we conducted principal components analysis (PCA; function glPca) and discriminant analysis of principal components (DAPC; function dapc) with the ADEGENET package in RStudio [v 2.0] [43,44]. DAPC was performed to identify the optimal number of genetic clusters based on the minimum Bayesian information criterion (BIC). Genetic differentiation was quantified using Weir and Cockerham’s pairwise F_ST_ estimator, inbreeding coefficients (F_IS_), observed heterozygosity (H_O_), and expected heterozygosity (H_E_) across all loci using the hierfstat package [v 0.5-11] [45]. To evaluate sensitivity to unequal sampling (MBMU *n* = 49, CBMU *n* = 27), all diversity and differentiation metrics were re-estimated after random downsampling to equal sample sizes (*n* = 27 per unit) using PLINK v1.9 (https://www.cog-genomics.org/plink/ accessed on 21 September 2026) (--thin-indiv-count), with results compared to full-sample estimates for robustness. All *p*-values were adjusted via Holm correction, and analyses were repeated using genetic cluster assignments as populations [46].

Contemporary migration rates were estimated using BA3-SNPs [v 1.1] [47] based on a random subset of 10,000 genome-wide SNPs. Analyses were conducted for the genetic subclusters and separately for a mountain-only dataset with the two MBMU subclusters. To assess convergence, the three-subcluster analysis was repeated across five independent chains with different random seeds, each using 500,000 burn-in iterations followed by 3,000,000 MCMC iterations sampled every 1000 iterations. Trace files were visually inspected for stationarity and mixing, and posterior means were compared across independent chains using the among-chain range and standard deviation for each migration parameter. Posterior uncertainty was evaluated from the 95% credible intervals of migration estimates. A mountain-only sensitivity analysis was used to evaluate whether estimates of recent exchange between MBMU subclusters were consistent when the CBMU cluster was excluded.

### 2.5. Inbreeding and Demographic History

H_O_, H_E_, and F_IS_ were calculated on a per-locus basis within each MU. The SNP locus was therefore the observational unit for statistical comparisons between MUs. Locus-specific estimates were compared between CBMU and MBMU using paired *t*-tests. Within each MU, paired *t*-tests were used to compare H_O_ and H_E_ with values paired by locus. Analyses were repeated separately for males and females to assess sex-biased dispersal.

Runs of homozygosity (ROH) were analyzed to evaluate patterns of autozygosity and to distinguish signals consistent with recent inbreeding from older or more diffuse shared ancestry. ROH were identified using PLINK v1.9 with the --homozyg function. The original analysis required a minimum ROH length of 500 kb (--homozyg-kb 500), at least 50 SNPs per segment (--homozyg-snp 50), and no heterozygous calls within a sliding window (--homozyg-window-het 0). The SNP-density threshold (--homozyg-density 5000) and maximum inter-SNP gap (--homozyg-gap 1000) were intentionally set to permissive values so that these criteria did not strongly constrain ROH detection, as described in Sams and Boyko (2018) [48]. Given that ROH inference can be sensitive to parameter choice, ROH calling was repeated across a focused set of alternative settings that varied the minimum ROH length, SNP support, SNP density, inter-SNP gap, and heterozygote tolerance. Full parameter settings and sensitivity results are provided in Supplementary Table S2 and Figure S1. For each bear, the number of ROH, total ROH length, mean ROH length, maximum ROH length, and F_ROH_, defined as the proportion of the genome in ROH, were calculated. ROH were also summarized by length class to distinguish short ROH, which may reflect older shared ancestry or background relatedness, from longer ROH, which are more informative of more recent autozygosity.

Contemporary effective population size (N_e_) was estimated using a linkage-disequilibrium based approach with NeEstimator [v.2.0.1] [49,50,51], which reflects the effective number of breeders contributing to recent generations. A MAF threshold of 0.05 was selected following the recommendations for large SNP datasets to reduce bias associated with rare alleles [50]. Estimates were generated for each MU and for genetically inferred clusters to evaluate differences in recent demographic processes and drift intensity. Because LD-based N_e_ estimates are sensitive to departures from panmixia, results were interpreted in conjunction with heterozygosity, inbreeding coefficients, and population structure.

Genome-wide allele frequency distributions were summarized for each genetic cluster. Given that SNPs below the applied minor allele frequency threshold had been removed during variant filtering, these distributions were interpreted descriptively rather than as an unfiltered site-frequency spectrum suitable for formal demographic inference. Relative differences among clusters were examined to assess whether allele-frequency patterns were broadly similar or showed evidence of greater drift within MUs.

### 2.6. Landscape Context Data

To provide landscape context for the observed genomic patterns, we summarized broad differences in land cover and transportation infrastructure between the two MUs. These analyses were not intended as formal landscape-genetic models, but rather as contextual summaries to evaluate whether MUs differed in features that could plausibly influence dispersal, connectivity, and local mating structure.

State-maintained road and interstate data, along with county boundaries, were obtained from the North Carolina Department of Transportation database. Counties were assigned to bear MUs, and major roadways intersecting each unit were summarized to characterize broad differences in transportation infrastructure. Land-cover data were obtained from the National Land Cover Database using the EVA tool from the Multi-Resolution Land Characteristics Consortium for the years 1985–2024 [52,53]. We focused on land-cover categories most relevant for black bear movement and habitat connectivity including total forest cover, net forest change, developed land, wetlands, and cultivated or agricultural land. Regional differences between MUs were evaluated using two-sided Wilcoxon rank-sum tests because proportional land-cover change variables were non-normally distributed [54]. Exact *p*-values were calculated with continuity correction disabled and were adjusted for multiple testing using the Holm method.

### 2.7. Targeted SNP Panel Development and Evaluation

To develop a candidate targeted SNP panel for black bear population assignment and individualization, the mPCRselect pipeline [55] was followed with the following filters: minimum genotype quality (MinGQ = 20), maximum individual missingness (IndvMissing = 0.5), minimum minor allele frequency (minMAF = 0.05), thinning distance (thin = 500,000), minimum nucleotide diversity (minPi = 0.1), minimum F_ST_ (minFST = 0.1), and linkage disequilibrium pruning (plinkLD_indep_pairwise = 500 kb, r^2^ = 0.20).

For population assignment, SNPs with the highest F_ST_ values between management units were selected using the Ruby script ‘get_best_snps.rb’ to identify top-ranked SNPs across simulation replicates. For individualization, SNPs with the highest nucleotide diversity (π) within each population were selected using the same script. Random match probability (RMP) was calculated for both populations using the R script ‘RMP_calc.R’. All scripts were adapted from Armstrong et al. [55]. This was repeated using the genetic cluster assignments to identify SNPs that would capture differences between genetic clusters. Resulting SNPs were combined to generate a comprehensive targeted panel after removing duplicates.

To evaluate the targeted panel, SNP subsets corresponding to the panel were extracted from the full dataset using PLINK (--extract). Population structure analyses (PCA, DAPC, clustering), genetic diversity metrics (H_O_, H_E_, F_IS_), F_ST_ calculations, and inbreeding analyses were then performed on each dataset using the same analytical pipeline.

Genotypes at loci included in the targeted SNP panel were extracted for the six individuals excluded from the genome-wide dataset during earlier filtering. Two individuals had been excluded from earlier analyses because they were identified as first-degree relatives of retained individuals, whereas four were excluded because they exceeded the genome-wide missing-data threshold of 40% (average missing call rate = 0.572). These genotypes were then added to the targeted panel genotype dataset for the remaining 76 bears. PCA and DAPC were then performed on the combined dataset of 82 individuals to evaluate whether the population of origin of the six previously excluded individuals could be recovered using the targeted SNP panel.

Probability of identity was calculated using allele frequency-based estimators for both unrelated individuals (PID) and siblings (PID_sibs_) [56,57]. PID values were calculated for three marker sets: (1) the full SNP dataset derived from WGS, (2) the targeted SNP candidate panel, and (3) the previously generated UrsaPlex STR panel [37]. PID_sibs_ was additionally calculated for the targeted SNP panel to provide a more conservative estimate of individualization power when individuals may be closely related. For independent loci, multilocus PID and PID_sibs_ were computed as the products of their respective single-locus probabilities across loci. Because combined PID values for large SNP panels become exceedingly small, calculations were performed on the log_10_ scale to maintain numerical precision (log_10_(PID_total) = Σ log_10_(PID_locus)).

## 3. Results

### 3.1. Whole-Genome Sequencing

**Figure 2:**
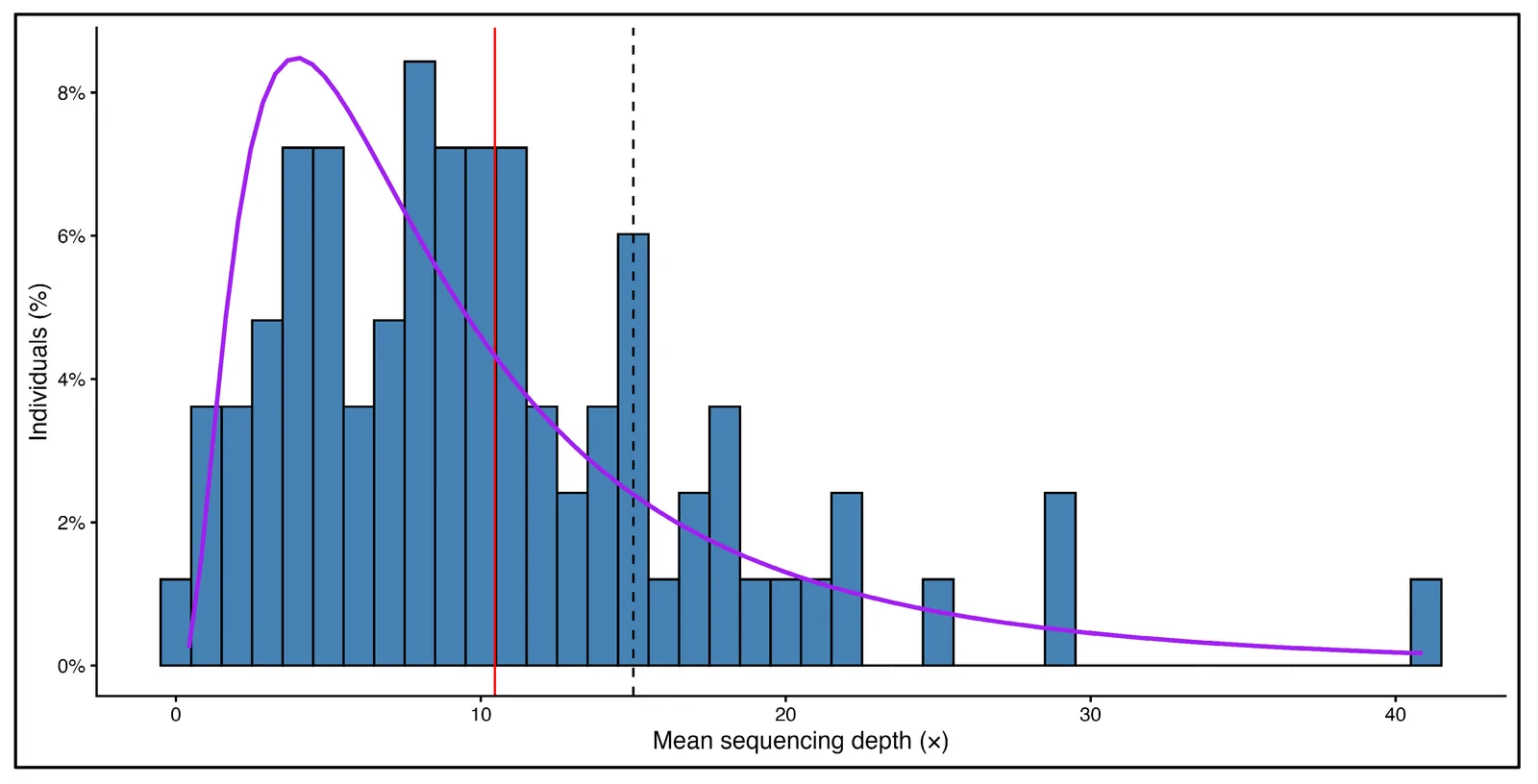
Distribution of mean sequencing depth across individuals. Histogram showing the distribution of mean sequencing depth (x) across all WGS individuals, shown as the percentage of samples per bin. The observed right-skewed distribution suggests moderate variability in sequencing depth among individuals, with most samples achieving sufficient coverage for downstream analyses. The purple curve represents a fitted density estimate. The red vertical line indicates the mean sequencing depth, while the dashed black line marks the 15x threshold targeted.

Following adapter trimming and quality filtering, reads exhibited high base quality, with an average of 95.6% of bases exceeding Q30 (range: 94–97%). Libraries yielded a mean of 118.1 million paired-end reads per individual (range: 18.0–420.1 million). Mean mapping rate to the reference genome was 94.3% (range: 20.2–99.9%), with 93.6% of reads properly paired. Duplicate reads accounted for 13.0% of reads on average, although the distribution was right skewed (median 11.0%; range: 8.1–55.5%), with most individuals exhibiting duplicate rates near 10–13% and a small number of samples showing substantially higher values. Duplicate rate was not associated with mean sequencing depth (Spearman’s rho = −0.048, *p* = 0.667), indicating that the higher duplicate fractions observed in some libraries were not explained by increased sequencing coverage. GC content distributions were consistent across samples (mean: 43.5%, range: 41.0–54.0%). Mean sequencing depth across all individuals was 10.5x (±7.2 SD), with most samples falling between ~5x and 15x (Figure 2).

Despite overall high sequencing quality, four individuals exhibited reduced sequencing performance relative to the dataset, characterized by lower mapping rates (mean: 50.7%), elevated duplicate read proportions (28.9%), and markedly lower mean coverage (0.82x). These individuals also produced fewer reads on average (72.1 million vs. 120.5 million in the other 78 samples). As a result, these four individuals were removed during downstream filtering for missing genotype data, yielding a dataset of 78 individuals (Table 1; see Supplementary Table S1 for details of the excluded samples).

For the remaining 78 individuals, sequencing metrics remained consistently high. Mean mapping rate was 96.5%, with 95.9% of reads properly paired. Mean duplicate read proportion was 12.2%, GC content averaged 43.35%, and mean sequencing depth was 10.95x, supporting robust genome-wide variant discovery and downstream population genomic inference. The transition-to-transversion ratio was 2.34, the homozygous-to-heterozygous genotype ratio was 0.88, and the insertion-to-deletion ratio was 0.77, indicating a balanced variant representation without evidence of systematic calling bias. Together, these metrics support the reliability of downstream population genomic analyses.

### 3.2. Variant Calling and SNP Selection

Across the dataset, 20.1% of loci deviated from the Hardy–Weinberg equilibrium at a nominal threshold (*p* < 0.05), with 2.53% exhibiting stronger deviation (*p* < 1 × 10^−6^). Examination of the direction of deviation revealed that 80.6% of loci significant at the nominal threshold exhibited a heterozygote deficit, consistent with a substantial contribution of population subdivision to the pooled signal. In contrast, among loci showing the strongest departures from equilibrium, 95.1% exhibited heterozygote excess, frequently at very high levels of observed heterozygosity. These extreme heterozygote-excess loci were also concentrated within particular scaffolds. Among the 644 scaffolds containing ≥50 SNPs, 35.6% had more than 10% of loci showing strong heterozygote excess, 17.5% had more than 25%, and 2.0% had more than 50%. This pattern is consistent with a subset of variants occurring in duplicated, paralogous, or otherwise problematic genomic regions and suggests that not all strong HWE departures are attributable to biological population structure.

When HWE was evaluated within MUs, the overall proportion of deviating loci decreased substantially (CBMU: 11.3% and 0.40%; MBMU: 7.5% and 2.16% for nominal and stronger thresholds, respectively). Most nominally significant deviations were characterized by heterozygote deficiency, whereas stronger deviations persisted at a smaller subset of loci, particularly within the MBMU, which may reflect a combination of previously identified substructure within this MU and locus-specific technical effects associated with regions exhibiting extreme heterozygote excess.

Two sets of first-degree relatives were identified, and one of each pair was randomly selected and removed, leaving 76 WGS datasets for all subsequent analyses. With 76 individuals retained (MBMU *n* = 49, CBMU *n* = 27), LD-pruning (--indep-pairwise 50 5 0.5) reduced the total number of SNPs for downstream analysis to 877,132 SNPs (877K). These filtering steps reduced potential biases from missing data, relatedness, and marker redundancy while preserving genome-wide variation suitable for population genetic inference.

### 3.3. Population Structure and Differentiation

**Figure 3:**
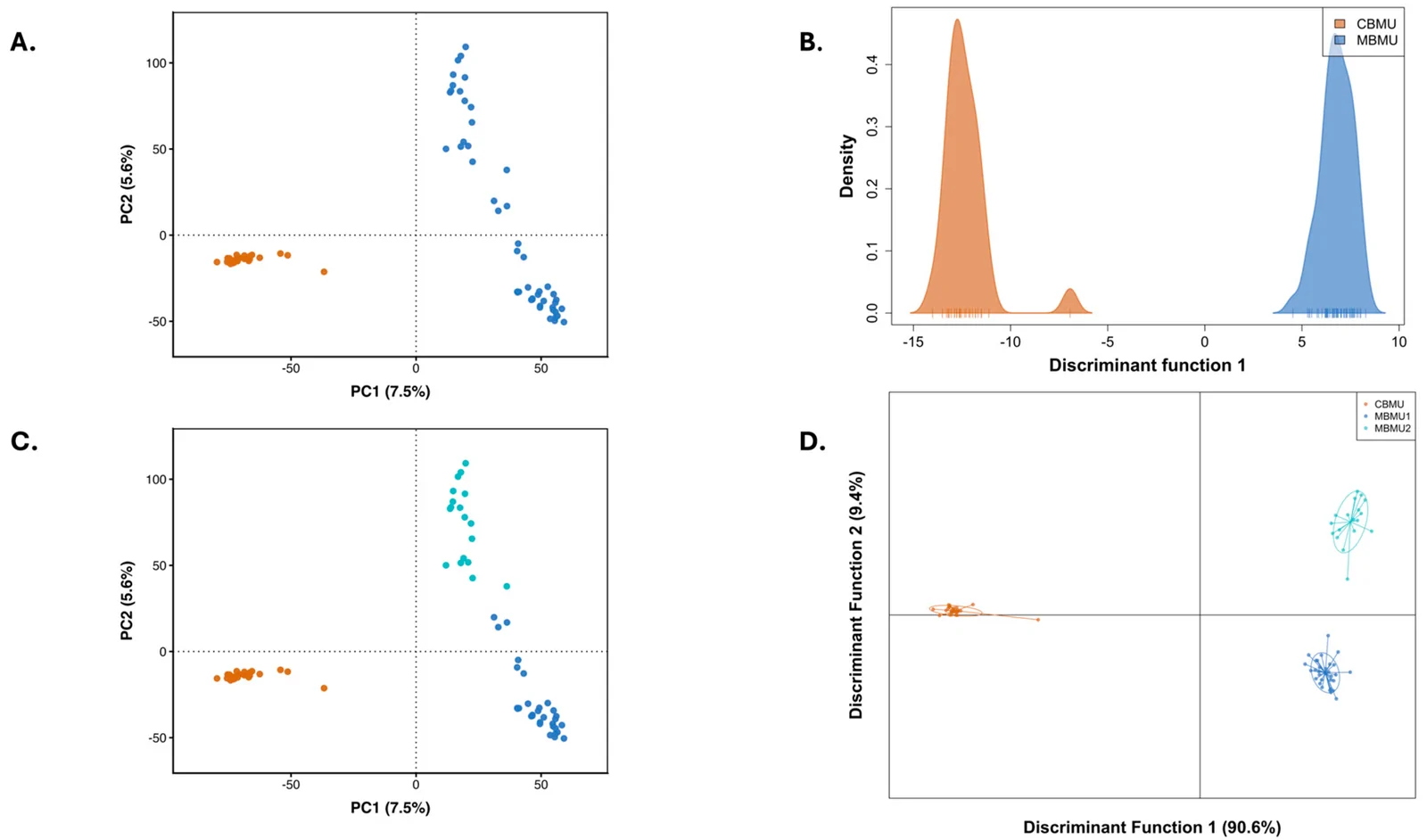
Genetic differentiation using the full 877K SNP dataset showing distinct separation between MUs and genetic clusters. (**A**) PCA using management unit assignments. (**B**) DAPC using management unit assignments. (**C**) PCA using genetic cluster assignments. (**D**) DAPC using genetic cluster assignments.

**Figure 4:**
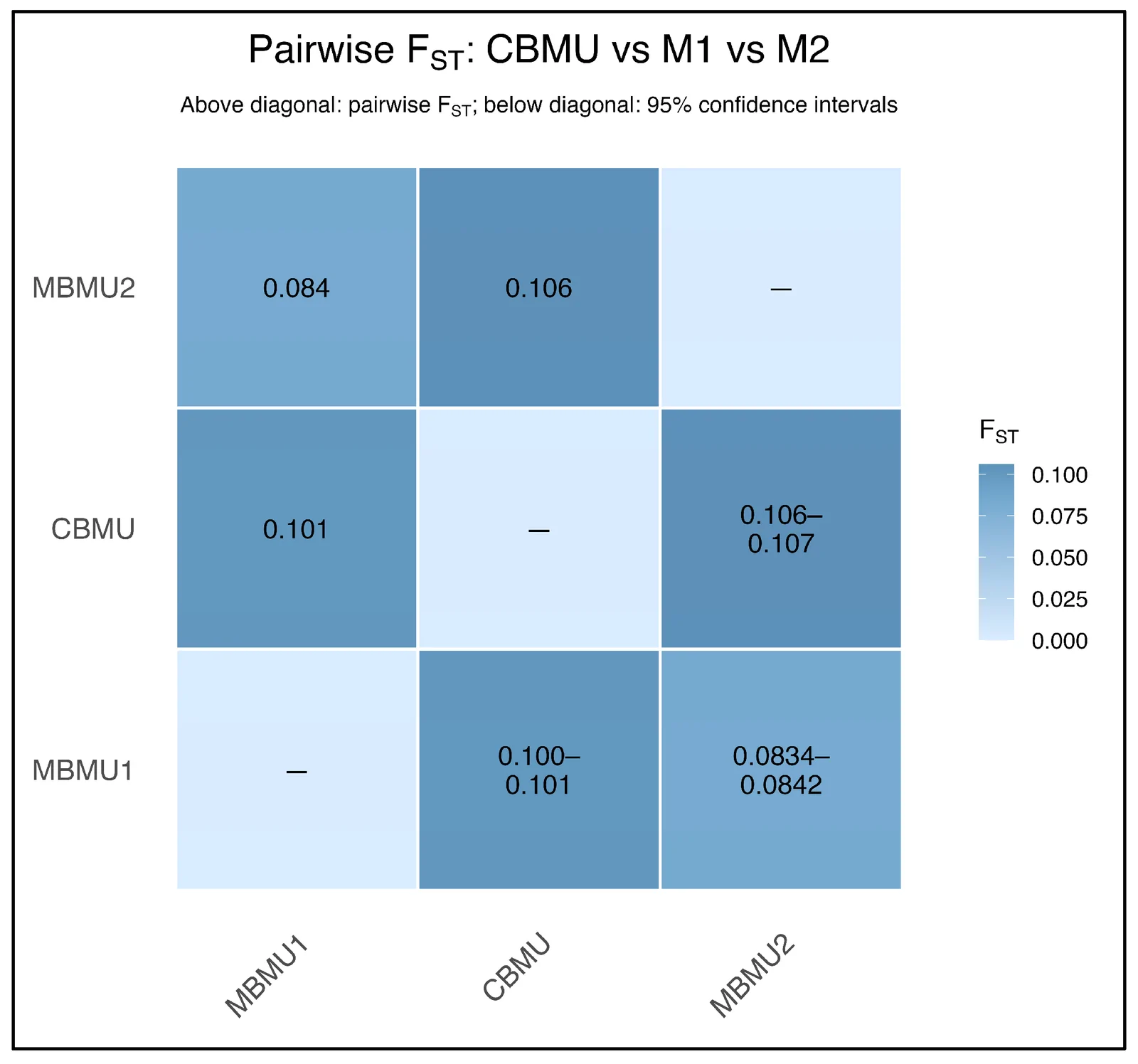
Pairwise F_ST_ between genetic clusters. F_ST_ values were calculated using the Weir and Cockerham estimator as implemented in the pairwise. WCfst function in the R package hierfstat. Values reflect the proportion of genetic variance attributable to population structure, with higher values indicating greater differentiation.

Genome-wide SNP analyses revealed clear genetic differentiation between the MBMU and CBMU (overall F_ST_ = 0.067), indicating moderate but biologically meaningful structure. Multivariate analyses (PCA and DAPC) consistently separated individuals by MU, with assignments matching the sample metadata (Figure 3A,B). All diversity and differentiation metrics were re-estimated after random downsampling to equal sample sizes (*n* = 27 per MU), yielding quantitatively identical results, confirming that observed patterns are not artifacts of uneven sampling (Supplementary Table S3). Within the MBMU, clustering analyses identified two partially overlapping genetic subclusters (M1: *n* = 31; M2: *n* = 18), indicating detectable internal structure without complete demographic independence. For individuals with available metadata, the spatial distribution of individuals assigned to M1 and M2 revealed geographic overlap, suggesting that genetic substructure reflects demographic processes (e.g., historical isolation or partial admixture) rather than discrete spatial segregation (Figure 3C,D). Pairwise differentiation (F_ST_) between MBMU clusters was 0.0838 (95% CI: 0.0834 to 0.0842), while differentiation between each MBMU cluster and the CBMU ranged from 0.101 to 0.106 (Figure 4).

**Figure 5:**
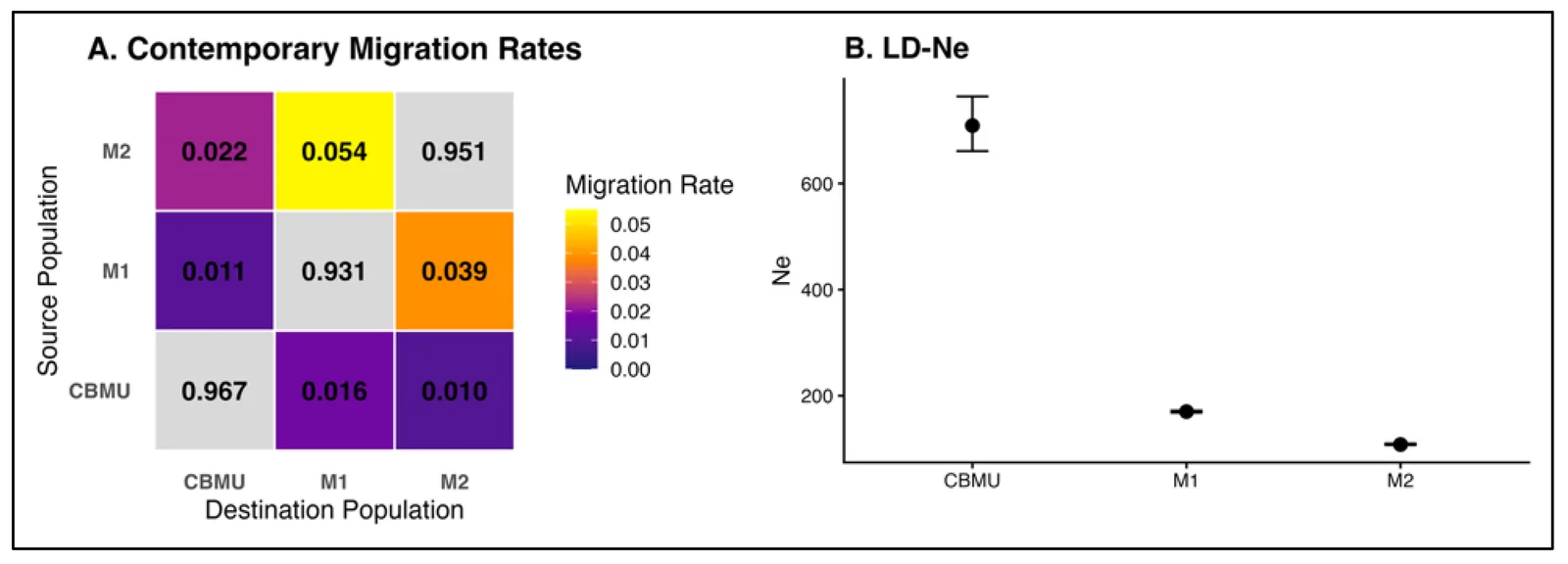
Contemporary gene flow and effective population size among NC black bear management units. (**A**) Contemporary migration rates among populations estimated using BA3-SNPs. Values represent the proportion of individuals in each destination population derived from each source population. Diagonal values indicate self-recruitment (non-migrants), while off-diagonal values represent migration. High self-recruitment (>0.93) across all populations and low asymmetric migration rates indicate limited recent gene flow among management units, with somewhat higher estimated genetic migration between M1 and M2 and lower estimated migration between CBMU and MBMU. (**B**) Linkage disequilibrium-based estimates of effective population size (LD-N_e_) for the CBMU and MBMU subpopulations (M1, M2) using NeEstimator. Points represent point estimates and error bars indicate confidence intervals.

BA3-SNP analyses indicated high self-recruitment within each inferred subcluster, with mean posterior estimates of 0.967 for the CBMU, 0.931 for M1, and 0.951 for M2 across five independent chains (Figure 5A). Estimated recent migration rates among subclusters were low overall, ranging from 0.010 to 0.054. Directional migration estimates between the CBMU and mountain subclusters were highly consistent across chains, with posterior means ranging from 0.010 to 0.022 and little among-chain variation. Estimates between the two MBMU subclusters were somewhat higher but differed in convergence among directions. M1 → M2 was highly consistent across chains (mean = 0.039; among-chain range = 0.038–0.040), whereas M2 → M1 showed greater among-chain variation (mean = 0.054; range = 0.047–0.071). The mountain only sensitivity analysis produced a similar overall pattern. M1 → M2 remained highly stable across chains (mean = 0.040; range = 0.0401–0.0409), whereas M2 → M1 showed greater variation (mean = 0.044; range = 0.0339–0.0497) and relatively broad posterior intervals. Thus, the analyses consistently supported high self-recruitment and low recent genetic migration, while the precise magnitude of M2 → M1 migration was less well-resolved. These results support limited recent genetic migration between MUs relative to that estimated between subclusters within the MBMU, but should not be interpreted as direct observations of individual bear movement.

### 3.4. Inbreeding and Demographic History

The CBMU exhibited significantly reduced observed heterozygosity relative to the MBMU (H_O_: 0.134 versus 0.158; mean difference = −0.0239; 95% CI: −0.0242 to −0.0237; paired t = −168.97; *p* < 2.2 × 10^−16^) and markedly elevated inbreeding coefficients (F_IS_: 0.136 versus 0.007; mean difference = 0.137; 95% CI: 0.136 to 0.138; paired t = 307.79, *p* < 2.2 × 10^−16^). Expected heterozygosity did not differ significantly between units (H_E_: 0.153 versus 0.154; mean difference = −2.96 × 10^−5^; 95% CI: −0.000306 to 0.000247; paired t = −0.21; *p* = 0.834), producing a pronounced heterozygote deficit in the CBMU (H_O_ < H_E_) and heterozygote excess in the MBMU (H_O_ > H_E_) (CBMU: mean difference = −0.0198; 95% CI: −0.0199 to −0.0196; paired t = −212.46; *p* < 2.2 × 10^−16^; MBMU: mean difference = 0.00413; 95% CI: 0.00396 to 0.00431; paired t = 48.20; *p* < 2.2 × 10^−16^). Within the MBMU, clusters differed significantly in diversity and inbreeding. M1 exhibited higher heterozygosity and lower F_IS_ than M2, indicating internal demographic heterogeneity (Table 2).

Sex-specific analyses revealed greater differentiation among females (*n* = 25; F_ST_ = 0.084; 95% CI: 0.083 to 0.086) than males (*n* = 51; F_ST_ = 0.063; 95% CI: 0.062 to 0.064). However, both sexes in the CBMU exhibited significantly reduced heterozygosity relative to their MBMU counterparts (females: mean difference = −0.0286; 95% CI: −0.0298 to −0.0275; paired t = −49.71 *p* < 2.2 × 10^−16^; males: mean difference = −0.0229; 95% CI: −0.0238 to −0.0221; paired t = −52.53; *p* < 2.2 × 10^−16^) and elevated inbreeding (females: mean difference = 0.155; 95% CI: 0.150 to 0.160; paired t = 63.443; *p* < 2.2 × 10^−16^; males: mean difference = 0.123; 95% CI: 0.120 to 0.126; paired t = 83.54, *p* < 2.2 × 10^−16^). Together, these patterns are consistent with sex-biased dispersal but suggest that male-mediated gene flow has not fully homogenized allele frequencies between the management units.

LD-based estimates of effective population size (N_e_) revealed contrasting demographic signals. Despite reduced heterozygosity and elevated inbreeding, the CBMU exhibited a substantially higher LD-N_e_ (mean N_e_ ~ 505) than both MBMU genetic clusters (M1: mean N_e_ ~ 180; M2: mean N_e_ ~ 109) (Figure 5B).

**Figure 6:**
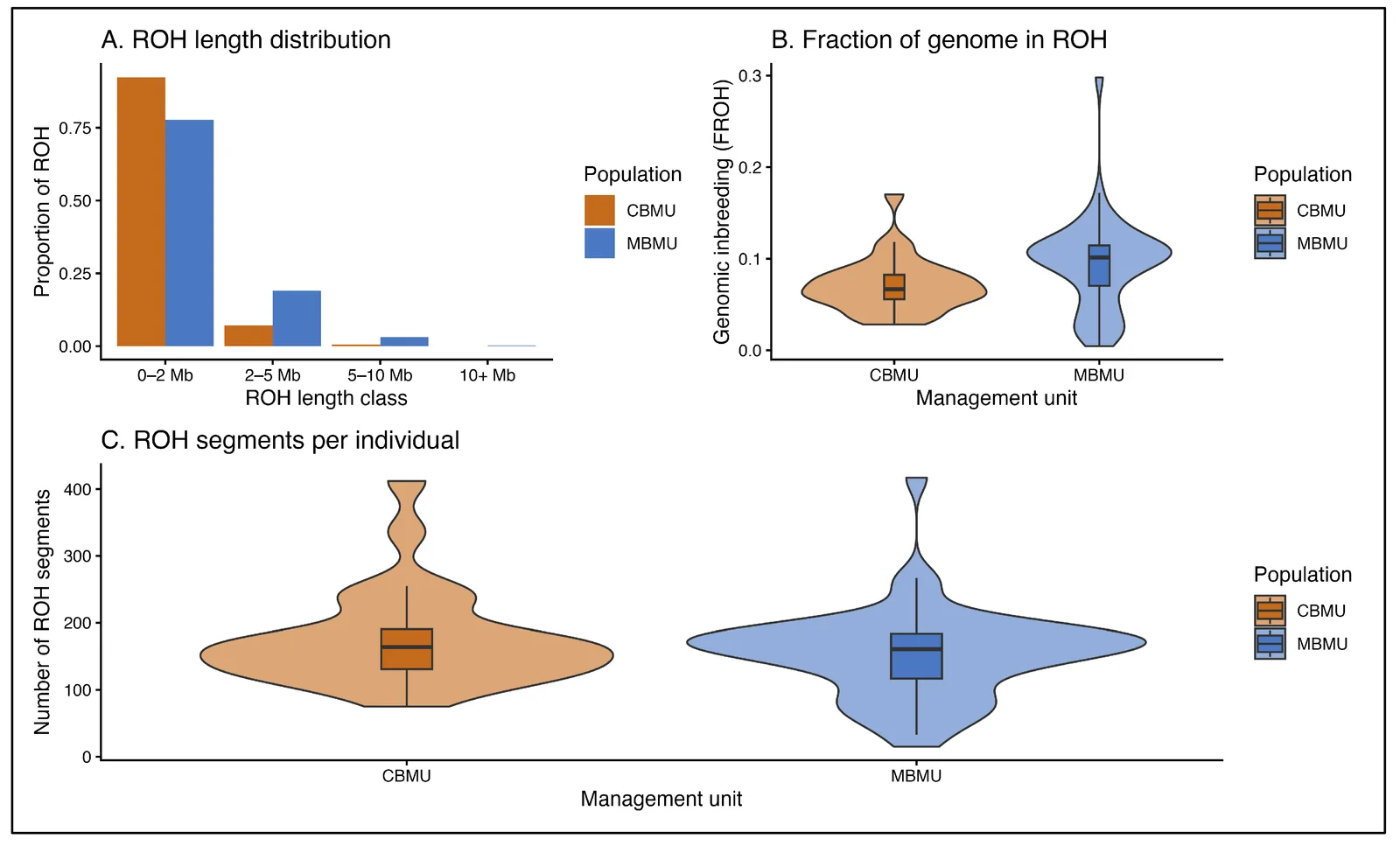
Runs of homozygosity revealed contrasting inbreeding patterns among management units. (**A**) ROH length distributions showed that the CBMU was dominated by short tracts (0–2 Mb), while the MBMU exhibited a greater proportion of longer ROH (>2 Mb). (**B**) The proportion of the genome in ROH (F_ROH_) was higher in the MBMU. (**C**) The number of ROH segments per individual was similar between management units and did not differ significantly between the CBMU and MBMU.

ROH analyses further clarified differences in inbreeding patterns (Figure 6). CBMU individuals exhibited significantly shorter mean ROH lengths (1022 kb vs. 1452 kb in MBMU; *p* = 6.35 × 10^−12^) (Figure 6A), while the number of ROH segments did not differ significantly between units. In contrast, MBMU individuals exhibited greater total ROH coverage (F_ROH_) and longer ROH tracts, including a higher proportion of segments >5 Mb, consistent with more recent or localized autozygosity (Figure 6A,B). Although absolute F_ROH_ estimates varied across parameter settings, the relative differences between MUs were consistent across the sensitivity analyses, with MBMU individuals exhibiting higher ROH burden than CBMU individuals. Because the biological interpretation was unchanged across sensitivity analyses, values from the original parameter set are reported here, with full sensitivity results provided in the Supplementary Materials (Supplementary Table S2 and Figure S1). Notably, this pattern contrasted with the population-level F_IS_ estimates. Although the CBMU exhibited significantly higher F_IS_, MBMU individuals exhibited greater F_ROH_ and longer ROH tracts.

Genome-wide allele frequency distributions derived from the 877K SNPs were broadly L-shaped across all clusters within the range of variants retained after filtering. The overall distributions were similar among groups, although M2 showed a relative reduction in lower-frequency variants compared with M1. Because the dataset had been filtered by minor allele frequency, these distributions were interpreted descriptively and were not used as independent evidence for or against a recent bottleneck. The relative shift observed in M2 is consistent with greater genetic drift within that subpopulation but should be interpreted cautiously given the filtering applied to the dataset.

Together, LD-N_e_, allele frequency spectra, and ROH patterns indicate that CBMU inbreeding reflects long-term restricted dispersal and diffuse autozygosity rather than recent demographic collapse, whereas the MBMU contains subpopulations experiencing more recent or episodic inbreeding.

### 3.5. Landscape Context Data

Landscape summaries showed broad differences between the two MUs that provide context for the genomic patterns observed in this study. CBMU counties experienced greater overall land-cover change than MBMU counties and retained substantially less forest cover than the mountain region. Although both units experienced forest loss, net forest decline was greater in the CBMU, where forested habitat occurs within a more fragmented matrix of agriculture, wetlands, development, and transportation infrastructure. CBMU counties also had a higher proportion of agricultural land, while MBMU counties retained greater total forest cover and more extensive contiguous forested habitat extending into neighboring regions.

**Figure 7:**
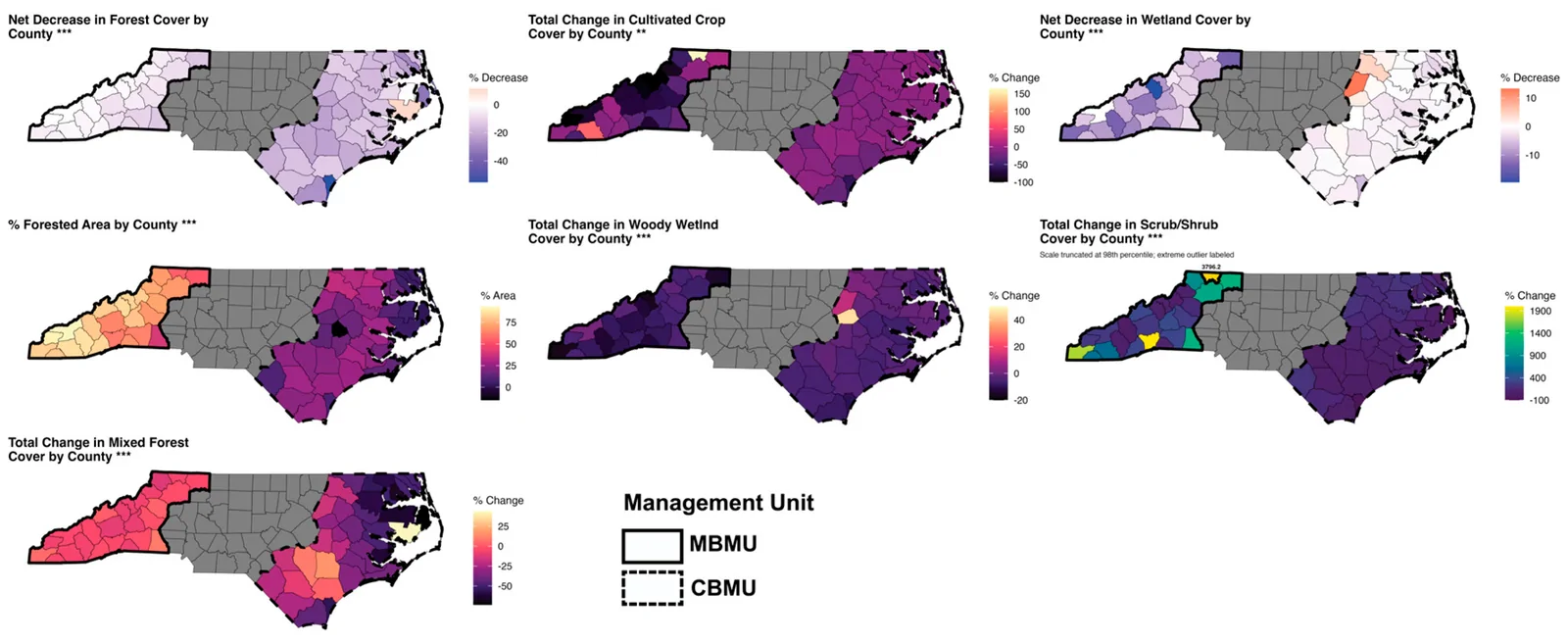
Land-cover and land-use variables that differed significantly between MUs. County-level variation in land-cover composition and land-use change across NC is shown for the CBMU (dashed boundary) and MBMU (solid boundary). Each panel represents an environmental variable derived from publicly available county-level data spanning 1985–2024. Only variables with statistically significant differences between the CBMU and MBMU are included. Color scales represent either percent change, net decrease, or proportional area. For variables with extreme values, color scales were truncated at the 98th percentile, and outlier counties were highlighted and labeled to preserve interpretability. Panel titles include adjusted significance annotations: *p* < 0.01 (**); *p* < 0.001 (***).

Several land-cover variables differed significantly between MUs, including total forest cover, net forest loss, agricultural land cover, and selected wetland or forest classes (Figure 7). In contrast, developed land, grassland, and net cultivated crop loss did not differ significantly between regions. Full land-cover summaries and statistical comparisons are provided in Supplementary Table S4. These landscape differences occurred alongside rapid human population growth, particularly in Piedmont and coastal counties. Because our genomic analyses identified strong differentiation between the two MUs, reduced diversity and elevated F_IS_ in the CBMU, and evidence of internal substructure within the MBMU, these landscape summaries provide a broad ecological context the observed genomic patterns and identify regional differences that are consistent with, but not directly demonstrate, constraints on dispersal and connectivity.

### 3.6. Targeted SNP Panel Development and Evaluation

A subset, targeted panel of 4990 SNPs was derived from the 877K dataset by retaining high-quality, biallelic loci, applying minor allele frequency and missingness filters, and pruning for linkage disequilibrium. SNPs were selected to ensure broad genomic distribution, yielding approximately one SNP per 506 kb of assembled sequence. SNP density scaled linearly with scaffold length (R^2^ = 0.963; r = 0.981; F_1_,_525_ = 13,720; *p* < 2 × 10^−16^) (Supplementary Figure S2A), indicating proportional genomic representation.

The targeted panel of 4990 SNPs was enriched for intermediate-frequency alleles relative to the full 877K dataset (mean MAF = 0.347 vs. 0.109), maximizing heterozygosity and individualization power. A two-sample Kolmogorov–Smirnov test confirmed a shift in allele-frequency distributions between the panel and genome-wide SNPs (D = 0.636, *p* < 2.2 × 10^−16^). Within management units, allele-frequency distributions showed modest shifts relative to the pooled dataset, consistent with population differentiation while maintaining substantial within-unit polymorphism. Pairwise F_ST_ estimates from the panel were highly concordant with genome-wide estimates (r^2^ = 0.9995), indicating that the targeted panel retained the major patterns of population differentiation observed in the genome-wide dataset (Supplementary Figure S2B).

Using only panel SNPs, PCA and DAPC recovered clear separation of CBMU and MBMU and subdivision within MBMU (Supplementary Figure S3). Overall F_ST_ increased to 0.124, reflecting enrichment for informative loci. Pairwise F_ST_ values among the three clusters ranged from 0.126 (M1 vs. M2) to 0.159 (M1 vs. CBMU).

The UrsaPlex STR panel yielded a log_10_ PID of −12.16 (PID ≈ 6 × 10^−13^). The 4990-SNP panel achieved a log_10_ PID value of −1835, while the full 877K dataset exceeded −113,000. For the targeted SNP panel, the log_10_ PID_sibs_ was −1126.86. PID accumulation curves showed that 19–31 SNPs would suffice for PID thresholds of 10^−9^, 10^−12^, and 10^−15^, matching/exceeding the UrsaPlex STR panel performance (Table 3).

**Figure 8:**
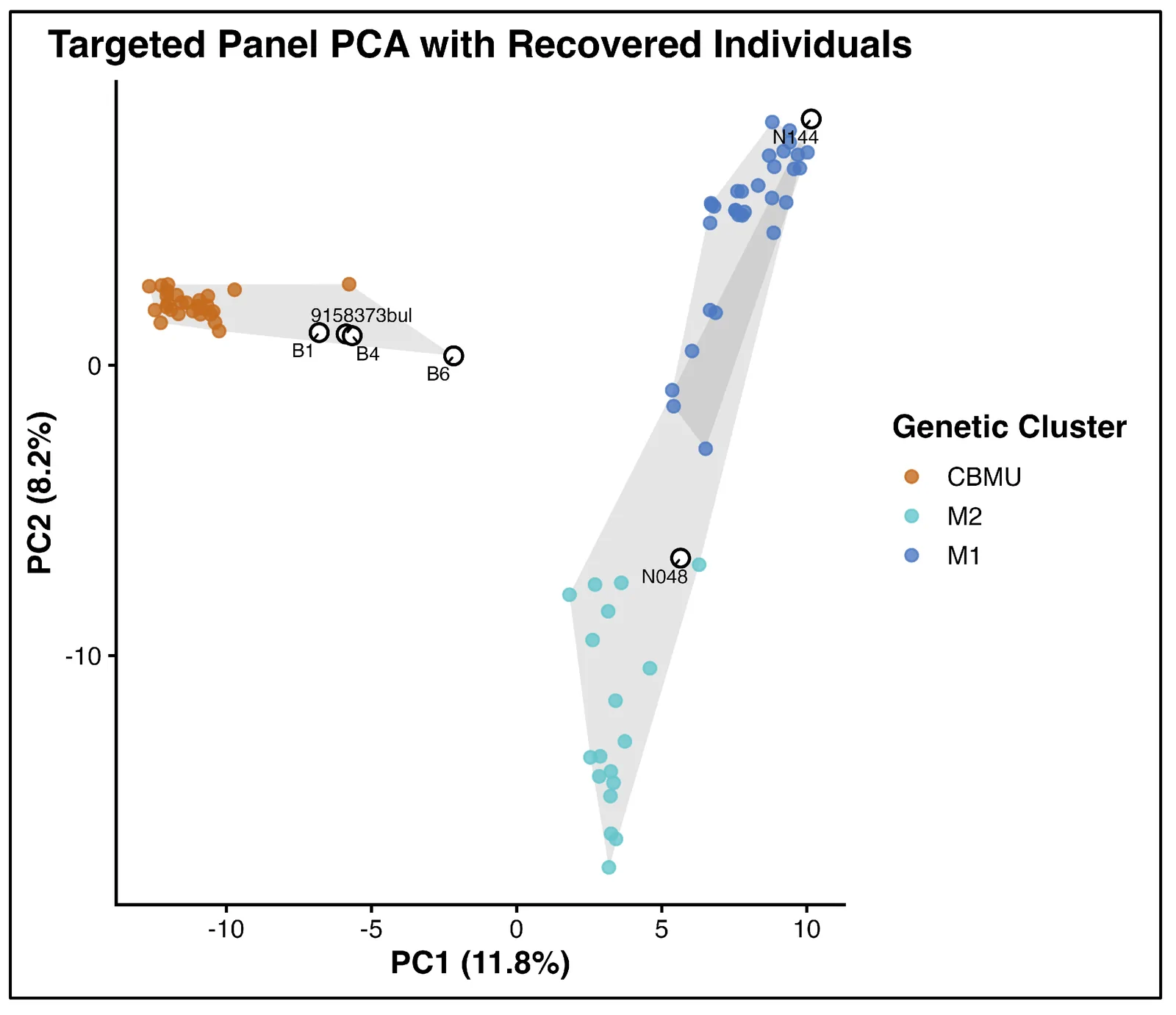
Population assignment of individuals removed during initial filtering using the targeted SNP panel. Principal component analysis (PCA) of individuals genotyped with the targeted SNP panel (4990 loci) including individuals previously removed during initial quality and relatedness filtering. For all six recovered individuals (labeled open circles), the correct MU assignment was recovered, and clear separation of genetic clusters was maintained, demonstrating the robustness of the candidate panel to missing data and its ability to recover accurate population structure.

To further evaluate panel performance, genotype data were recovered for the six individuals excluded during earlier filtering steps. Genotypes were extracted for the targeted SNP set and merged with the baseline dataset prior to multivariate analyses. Both DAPC and PCA analyses correctly assigned all six bears to their respective geographic populations (Figure 8). Four individuals originating from the CBMU clustered with the CBMU reference samples, while two individuals from MBMU grouped within the MBMU cluster and were distributed across the previously identified MBMU subclusters.

## 4. Discussion

Our whole-genome analysis of 76 black bears in North Carolina revealed three key findings. First, the MBMU and CBMU are genetically distinct, with additional substructure detected within the MBMU. Second, the CBMU has reduced genetic diversity and elevated inbreeding despite a larger estimated census size. Finally, these patterns are consistent with long-term restrictions on connectivity and occurred within contrasting geographic and landscape contexts.

Genome-wide F_ST_ and multivariate analyses revealed clear genetic differentiation between the CBMU and MBMU. This pattern is consistent with limited connectivity across the central Piedmont, where major urban centers, transportation corridors, and fragmented habitat separate coastal and mountain bear populations. Historical NCWRC range data show that while black bears have recolonized suitable habitats across much of North Carolina, range expansion has remained limited across the densely developed corridor encompassing the Raleigh-Durham-Chapel Hill, Greensboro-Winston-Salem, and Charlotte metropolitan areas [19]. Black bears exhibit strong aversion to crossing open, developed landscapes [15], and major roadways can reduce functional connectivity through both behavioral avoidance and vehicle collision [58,59]. Although our road and land-cover summaries do not directly estimate landscape resistance, they show that the two management units occupy markedly different landscapes. While formal landscape-genetic analyses are needed to identify specific barriers to gene flow, the stark differences in habitat provide a compelling landscape context for the genetic separation observed between the two MUs. The CBMU occurs within a more heavily modified coastal and agricultural matrix with substantially lower total forest cover, whereas the MBMU remains embedded within a more continuous Appalachian forest landscape with potential connections to neighboring states.

Sex-specific analyses further support the interpretation that landscape context influences connectivity between the MBMU and CBMU. Females showed greater differentiation between MUs than males, consistent with female philopatry and male-biased dispersal documented in this species [60,61,62,63,64]. Although less pronounced, males still retained measurable differentiation between management units. This pattern is compatible with greater male-mediated connectivity, but the overlap in genetic differentiation between sexes indicates that male dispersal has not completely homogenized allele frequencies between the two regions. Both sexes in the CBMU exhibited reduced heterozygosity and elevated F_IS_ relative to their MBMU counterparts, suggesting that the observed differences are not restricted to the more philopatric sex. The landscape separating the MBMU and CBMU may restrict even male-mediated gene flow, though the present analyses do not directly test the effects of specific landscape features on dispersal or gene flow. The heavily occupied landscape of the central Piedmont, characterized by high road density and a human population of more than 6.7 million people, likely reduces the frequency and success of long-distance dispersal events between the two MUs. Roads cause direct mortality through vehicle collisions, an important cause of adult bear death in many populations and can act as behavioral barriers that reduce gene flow even where occasional crossings occur. Cumulatively, these results suggest that functional connectivity between the MBMU and CBMU is insufficient to counteract genetic drift and maintain shared allele frequencies, despite the species’ intrinsic dispersal capacity.

Within the MBMU, clustering analyses identified two partially overlapping genetic subclusters, yet contemporary migration rates indicate ongoing demographic connectivity. This suggests that the MBMU is not panmictic, but neither does it appear to consist of fully independent populations. The observed substructure likely reflects a combination of historical recovery dynamics, incomplete mixing, natural topographic complexity, and ongoing movement through relatively continuous Appalachian habitat. The presence of heterozygote excess within the MBMU is consistent with recent admixture or secondary contact between historically differentiated lineages [65]. This interpretation is plausible given the geographic position of western NC within the broader Appalachian region, where forested habitats connect to bear populations in Tennessee, Georgia, and other neighboring states. These connections could introduce novel alleles and elevate heterozygosity above expectations for a single closed population.

Previous analyses using the UrsaPlex v2.0 STR panel identified broadly similar population structure [37]. Although that study was primarily designed to evaluate the panel for individual identification and forensic applications, ParallelSTRUCTURE supported three major genetic clusters, with CBMU bears forming a relatively cohesive group and MBMU bears separating into two more admixed clusters. DAPC similarly distinguished CBMU from MBMU bears and identified additional substructure within the MBMU, although the exact number of inferred MBMU subclusters varied among analyses. The STR-based DAPC also showed greater overlap among the MUs and among MBMU subclusters than observed in our genome-wide SNP analyses. This difference likely reflects the substantially greater marker density and genome-wide coverage of the SNP dataset, which provides greater resolution for detecting subtle allele-frequency differences among partially differentiated groups. Despite variation in the exact number and separation of inferred clusters, both studies consistently support stronger and more complex genetic subdivision within the MBMU than within the CBMU. The present study therefore corroborates the broader structure identified previously while providing a finer resolution of differentiation among groups and extending those findings through estimates of contemporary migration, N_e_, genomic diversity, and patterns of homozygosity.

Historical translocation practices may also have contributed to the overlapping genetic structure observed within the MBMU. For several decades, bears were translocated into western North Carolina from the Great Smoky Mountains National Park and supplemented by releases of captive-born individuals, while neighboring states have also relocated conflict bears near state borders. These movements, combined with the species’ high dispersal capacity, may have facilitated gene flow across the region and contributed to the admixture signals detected here. Although this study lacks samples from adjacent state populations, the genomic patterns suggest that bears from different geographic or historical source areas may be interbreeding within western NC. The MBMU appears to reflect a system shaped by both natural dispersal and management-mediated movement, rather than strictly historical isolation or contemporary fragmentation.

Despite having a larger population size (~11,000 vs. ~8000 individuals), the CBMU exhibited reduced H_O_ and elevated F_IS_ relative to the MBMU. Initially, this appears inconsistent with its relatively high LD-N_e_ and retention of rare alleles. Reconciling this requires recognizing that different genetic metrics capture different aspects of population history and structure. Clustering methods primarily identify major axes of allele-frequency differentiation and can return a single genetic cluster even when finer-scale spatial structure, localized relatedness, or departures from random mating are present. In contrast, heterozygosity and F_IS_ can be sensitive to these finer-scale processes [66]. The CBMU illustrates this distinction, forming a single genetic cluster while showing heterozygote deficits and elevated F_IS_, indicating some departure from random mating. Fine-scale spatial structure or restricted dispersal among breeding neighborhoods could contribute to this pattern, and when individuals are sampled across a broad geographic area, could produce a Wahlund-like heterozygote deficit [67,68,69,70,71,72]. However, because we did not detect discrete genetic subdivisions within the CBMU, this is not definitive evidence of a Wahlund effect. For this reason, the CBMU was treated as a single population for LD-N_e_ estimation. However, if subtle spatial structure is present below the resolution of our clustering analyses, it could contribute to linkage disequilibrium and influence the resulting estimate.

The contrasting F_IS_ and F_ROH_ patterns further emphasize that these metrics capture different dimensions of inbreeding. F_IS_ reflects departures from expected heterozygosity at the population level and can be influenced by nonrandom mating or cryptic structure, while F_ROH_ measures autozygosity within individuals. Accordingly, the elevated F_IS_ in the CBMU does not necessarily imply greater recent autozygosity. The CBMU bears exhibited lower F_ROH_ and predominantly shorter ROH, while MBMU bears showed higher F_ROH_ and longer tracts despite low MU-level F_IS_. This suggests that the CBMU signal may reflect longer-term or fine-scale departures from random mating, whereas the MBMU pattern is more consistent with greater recent or localized shared ancestry within some individuals.

Interpretations of the allele-frequency distributions are limited by prior MAF filtering, but their broadly similar shape among groups, together with the ROH patterns, is consistent with the documented recovery of bear populations following substantial declines in the early 20th century. The predominance of short ROH segments and lack of a strong excess of long ROH segments across sensitivity analyses do not support widespread recent inbreeding. Short ROH are generally more representative of older shared ancestry, historical bottlenecks, long-term reductions in N_e_, or background relatedness, as recombination breaks ancestral homozygous tracts into shorter segments over time [73,74,75,76]. Instead, the heterozygote deficit, elevated F_IS_, and predominance of short ROH are consistent with long-term restrictions on connectivity or nonrandom mating operating at a finer spatial scale than was resolved by our clustering analyses, although these mechanisms cannot be distinguished directly with the present sampling design. In other systems, heterozygote deficits, short ROH, and elevated F_IS_ within an otherwise unstructured population have been signatures of ongoing, incomplete dispersal restriction without discrete subdivision [77,78,79]. Even in highly mobile species like black bears, behavioral or landscape restrictions can maintain fine-scale genetic structure without discrete subpopulations [80].

Although the data do not identify the specific mechanism underlying the CBMU heterozygote deficit, the contemporary landscape provides important context for how long-term restrictions on connectivity may be maintained or intensified. By 2024, CBMU counties had experienced substantially greater land-cover change than MBMU counties, retaining only approximately one-quarter of the forest cover present in the mountain region. Fragmentation of the forest-wetland-agriculture mosaic that CBMU bears depend on for movement and resources supports the genomic patterns suggestive of reduced connectivity [19,81]. Beyond internal fragmentation, the CBMU faces broader geographic constraints. The MU is bounded by the Atlantic coastline to the east, development along the state borders to the north and south and increasing urban growth at its inland edge. The remaining unoccupied habitat largely abuts areas of dense human population [19]. Collectively, coastal geography, limited interstate connectivity, and extensive urban development are consistent with reduced opportunities for immigration and dispersal and may contribute to a functional “peninsular effect” [82]. Continued loss of these habitats could potentially intensify these constraints even if the overall census size stays high [8,9,51,81,83]. As these landscape analyses are contextual rather than formal landscape–genetic tests, we do not interpret recent land-cover change as the primary cause of the older genomic signatures described above. Rather, these landscape changes represent contemporary pressures that may maintain or intensify restrictions on dispersal and genetic connectivity.

Together, the contrasting genomic and landscape patterns provide a framework for interpreting connectivity in NC black bears. The MBMU appears to benefit from broader-scale connectivity, potential interstate gene flow, and a more continuous Appalachian forest landscape, which may help support higher diversity and admixture signals despite internal substructure. In the CBMU, reduced diversity and elevated F_IS_ occur within a landscape characterized by greater fragmentation and geographic constraint and are consistent with long-term limits to connectivity.

The genome-wide dataset also provided an opportunity to evaluate how genomic data could be translated into more targeted monitoring tools. Comparison with the UrsaPlex STR panel demonstrated that relatively few highly informative SNPs are required for individual discrimination. Although UrsaPlex provides strong individual discrimination (PID = 6.9 × 10^−13^), accumulation analysis indicated that approximately 31 selected SNPs were sufficient to achieve comparable discriminatory power in the NC dataset.

The 4990-SNP dataset was developed specifically as a candidate resource to support the individual identification and assignment of bears to management units and major genetic clusters using a substantially reduced set of genome-wide markers. Accordingly, marker selection emphasized broad genomic distribution and population-informative polymorphisms rather than optimization for estimating genome-wide demographic parameters. The panel successfully retained the major patterns relevant to these intended applications, including discrimination between the CBMU and MBMU and resolution of the genetic clusters identified in the whole genome analyses. This dataset should be viewed as a candidate resource for individual identification, population and cluster assignment, capture–recapture applications, and related forensic or management uses, rather than as a substitute for WGS for the estimation of ROH, genomic inbreeding, demographic history, or other parameters that depend on dense and relatively uniform genome-wide marker coverage.

Consistent with this intended purpose, the targeted panel retained strong differentiation among the management units and major genetic groups. However, because loci were selected in part for their ability to distinguish these groups, the greater differentiation observed with the targeted panel relative to the genome-wide dataset is expected and likely reflects ascertainment toward highly differentiated loci. Accordingly, F_ST_ and other population-genetic estimates calculated from this marker set should not be interpreted as directly equivalent to genome-wide estimates or as evidence of greater underlying population differentiation. Rather, the panel was designed to retain loci informative for distinguishing the management units and genetic clusters identified in the genome-wide dataset.

The analysis therefore establishes proof-of-concept for a targeted marker resource for identification and assignment, but it does not constitute a laboratory-validated genotyping assay. Given that SNP selection and evaluation were conducted using the same dataset, the performance estimates reported here are not based on independent validation and may therefore overestimate assignment performance. Validation of these SNPs with an independent sample set would be an important next step if routine individual identification, population or genetic-cluster assignment, capture–recapture monitoring, or forensic genotyping becomes a management priority in North Carolina.

While this study establishes a framework for understanding population structure in NC’s black bears, it highlights directions for future research. Sampling was restricted to the MBMU and CBMU, but the incorporation of samples from the PBMU and neighboring states will be necessary to resolve broader population dynamics. Furthermore, while our land-cover and road summaries effectively illustrate the contrasting regional contexts of these management units, landscape–genetic analyses would strengthen the identification of barriers to gene flow. Because samples were collected opportunistically without precise geographic coordinates, we could not assess fine-scale spatial patterns. Future work integrating regional sampling, GPS telemetry, formal landscape–genetic models, and laboratory validation of a reduced SNP marker set will be essential to pinpoint specific movement corridors, evaluate localized barriers to gene flow, determine the relative contributions of historical demographics versus contemporary fragmentation, and develop a cost-effective and accessible resource for bear genotyping.

## 5. Conclusions—Conservation and Management Implications

Genome-wide data show that the population structure within North Carolina’s black bears is more complex than geographic management boundaries alone would suggest. The MBMU has two genetically distinguishable subclusters, but there is insufficient evidence to conclude that these subclusters function as fully independent populations. This distinction is important for management, as genetic substructure alone does not imply demographic independence [84]. The MBMU appears to have ongoing connectivity, but differences in diversity, N_e_, and ROH burden between subclusters suggest that localized vulnerabilities could emerge if connectivity is further reduced. Maintaining movement within the MBMU and across broader Appalachian corridors will be important for preserving genetic variation and preventing localized drift.

The CBMU forms a single genetic cluster but also shows signatures consistent with fine-scale spatial structure and restricted mating among local breeding neighborhoods including heterozygote deficits, short ROH, and elevated F_IS_. These patterns do not require discrete subpopulations; rather, they can arise under limited dispersal and recent or moderate habitat fragmentation within an otherwise broadly homogeneous unit. This interpretation is consistent with studies showing that fine-scale genetic structuring can emerge over short distances in fragmented landscapes, even when broader population boundaries are not [77,78,79]. Conservation strategies in the CBMU should therefore prioritize maintaining and restoring functional connectivity among forested and wetland-associated habitats, reducing road mortality, and protecting movement opportunities across increasingly developed landscapes.

Human–bear conflict intersects directly with genetic health. Conflict-related removals, vehicle collisions, and harvest can disproportionately affect dispersing and reproductively active individuals, potentially eroding connectivity and accelerating drift [85,86,87,88]. In the CBMU, more loss could intensify existing heterozygote deficits or increase local relatedness. In the MBMU, uneven removals could exacerbate subcluster differences. Integrating genomic data into harvest planning, conflict mitigation, translocation decisions, and long-term monitoring would allow managers to address immediate human–bear conflicts while also safeguarding the genetic diversity needed for long-term population resilience.

These results support a wildlife management approach that recognizes both broad-scale structure and fine-scale vulnerability. Management in the MBMU should avoid imposing artificial subdivision while continuing to monitor localized diversity and connectivity to prevent future fragmentation. In the CBMU, managers must account for the fact that a large census size does not guarantee genetic security. Because the genomic patterns are consistent with limited connectivity, conservation strategies should prioritize the protection of forest-wetland mosaics, mitigation of road mortality through targeted crossing structures or road-planning efforts, and the preservation of dispersal corridors that may help maintain gene flow within and between management units.

Collectively, our results show that genome-wide data can reveal population structure, demographic history, and emerging connectivity concerns that may not be apparent from census size or management boundaries alone. The MBMU appears genetically diverse and connected but internally structured, whereas the CBMU appears broadly homogeneous but genetically more vulnerable. Proactive habitat planning, barrier mitigation, and continued genomic monitoring will be important for maintaining the evolutionary viability of both North Carolina black bear management units, particularly as human population growth, land-use change, and transportation infrastructure continue to reshape the landscapes through which bears move.

## Figures and Tables

**Table 1:** Summary of bear samples used in the study. Post-filtering reflects the number of bears remaining in the dataset after removing individuals with missing genotype data and first-degree relatives. Full metadata are available in Supplementary Table S1.

Management Unit	Number of Bears	Number of Bears After QC Filtering	Number of Bears After Relatedness Filtering
CBMU	31	27	27
MBMU	51	51	49
Total	82	78	76

**Table 2:** Genetic diversity, inbreeding, and effective population size by management unit and genetic cluster. Data summarize the population genetic metrics for black bears at the management unit (CBMU, MBMU) and genetic cluster (M1, M2, CBMU) levels. Metrics include sample size (n), average observed (H_O_) and expected heterozygosity (H_E_), average inbreeding coefficient (F_IS_), runs of homozygosity (F_ROH_), and LD-based effective population size (N_e_). An LD-based N_e_ estimate is not reported for MBMU because it comprises multiple genetically distinct subclusters, violating the assumption of a single, panmictic population required for reliable LD-based estimation.

Comparison	Group	n	H_O_	H_E_	F_IS_	F_ROH_	N_e_
Management unit	CBMU	27	0.1342	0.1539	0.1357	0.0716	505.0
	MBMU	49	0.1581	0.1540	0.0069	0.0942	-
Genetic clusters	M1	31	0.1554	0.1473	−0.0122	0.0968	179.7
	M2	18	0.1628	0.1512	−0.0401	0.0896	108.7
	CBMU	27	0.1342	0.1539	0.1357	0.0716	505.0

**Table 3:** Number of SNPs required to achieve target probabilities of identity. The table shows the number of SNPs required to reach increasingly stringent probabilities of identity (PID) thresholds. PID represents the probability that two unrelated individuals share the same multilocus genotype by chance.

Target PID	SNPs Required
1.00 × 10^−9^	19
1.00 × 10^−12^	25
1.00 × 10^−15^	31

## Data Availability

The original contributions presented in this study are included in the article/Supplementary Materials. Further inquiries can be directed to the corresponding author.

## Supplementary Materials

The following supporting information can be downloaded at: https://www.mdpi.com/article/10.3390/genes17101206/s1. The supplementary dataset generated and analyzed during this study can be found in the Dryad repository (https://doi.org/10.5061/dryad.76hdr7tbk). The dataset includes a merged variant call format (VCF) file containing genome-wide single nucleotide polymorphisms (SNPs) for all individuals analyzed and a list of SNPs included in the targeted SNP panel. Table S1: Metadata for all individuals included in the dataset; Table S2: Parameters evaluated in the sensitivity analyses for runs of homozygosity (ROH) detection; Table S3: Downsampling analyses of genomic diversity and inbreeding. Table S4: Land cover change and composition across management units from 1985–2024; Figure S1: Sensitivity of ROH-based inbreeding estimates to PLINK parameter settings. F_ROH_ values were calculated across a series of ROH-calling parameter sets for all individuals. Figure S2: Validation and genomic representation of the targeted SNP panel; Figure S3: Genetic differentiation using the targeted 4990 SNP dataset.
