# Supplementary material for: Population Genomics of North Carolina’s Black Bears (Ursus americanus) Reveals Substructure and Identifies Informative SNPs for Population Assignment and Individualization
Source: Genes (Basel). 2026 Sep 29;17(10):1206. doi: 10.3390/genes17101206 (PMC13704591; doi:10.3390/genes17101206)

Article Title: Population Genomics of North Carolina's Black Bears (Ursus americanus) Reveals Substructure and Identifies Informative SNPs for Population Assignment and Individualization.

Supplementary Figure S1. Sensitivity of ROH-based inbreeding estimates to PLINK parameter settings. F_ROH values were calculated across a series of ROH-calling parameter sets for all individuals. The original reported setting used a 500 kb minimum ROH length, 50 SNP minimum, SNP-density threshold of one SNP per 5000 kb, maximum inter-SNP gap of 1000 kb, and zero heterozygous calls. The conservative comparison used the same minimum length and SNP threshold but a stricter SNP-density threshold of one SNP per 50 kb and maximum gap of 500 kb. Additional sensitivity analyses varied minimum ROH length, SNP support, SNP density, maximum inter-SNP gap, and heterozygote tolerance. **A)** Management unit comparison. **B)** Genetic cluster comparison.

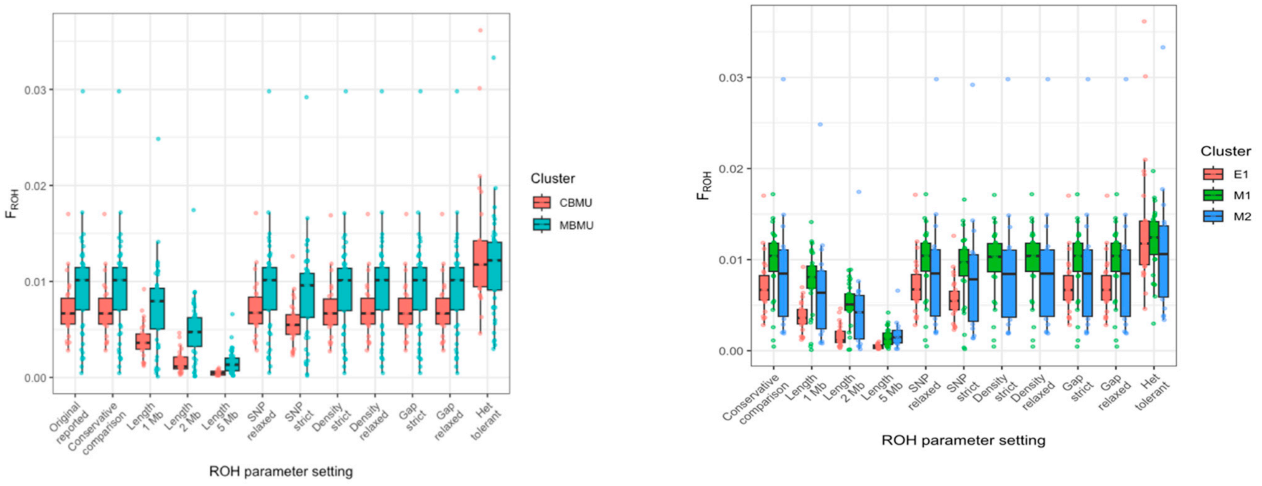

(A) **(B)**

Supplementary Figure S2. Validation and Genomic Representation of the Targeted SNP Panel. A) Relationship between scaffold length and SNP panel representation. The number of SNPs retained in the targeted panel scales linearly with scaffold length, demonstrating proportional and unbiased genomic coverage. The fitted regression line indicates consistent SNP density across scaffolds, supporting even genome-wide representation in the panel design. SNP panel markers were broadly distributed across the genome, covering ~94.5% of scaffolds with a median inter-marker spacing of ~323 kb. **B)** Concordance between genome-wide and targeted SNP panel estimates of genetic differentiation (FST). Pairwise FST values calculated from the full genome-wide (877K SNPs) and the reduced SNP panel (4,990 SNPs) show near perfect agreement ($R^2 = 0.9995$), indicating that the targeted panel fully recapitulates patterns of population structure captured by the genome-wide data

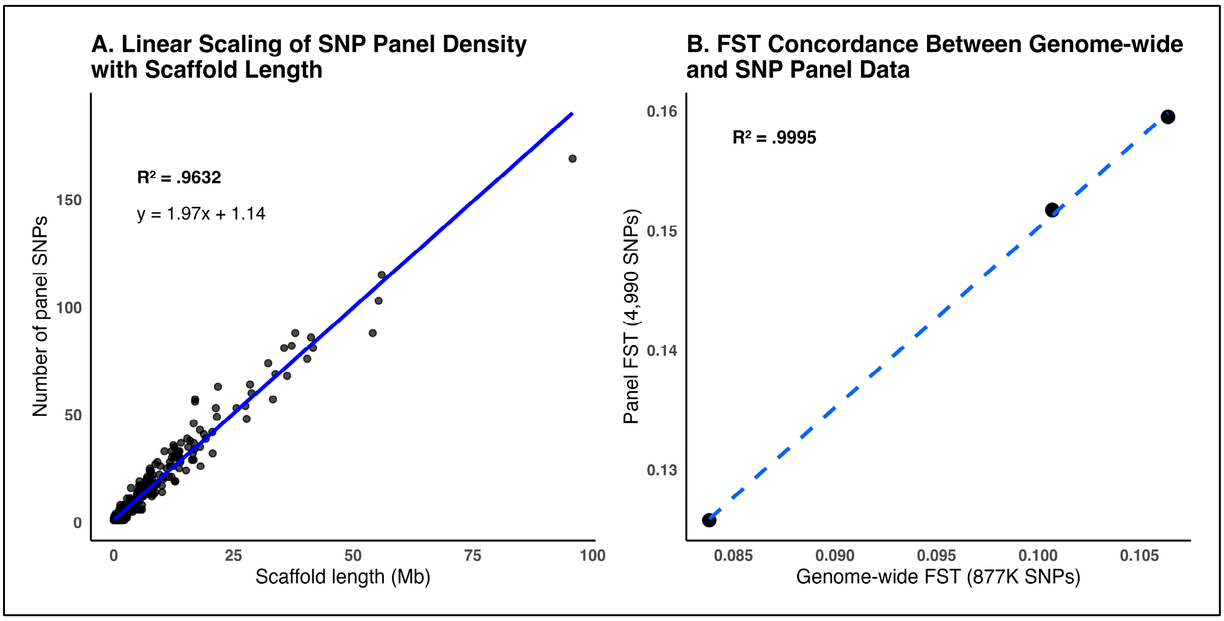

Supplementary Figure S3. Genetic differentiation using the targeted 4,990 SNP dataset. Results show distinct separation between MUs and genetic clusters and recapitulation of patterns seen with the 877K dataset. **A)** PCA using Management Unit assignments, **B)** DAPC using Management Unit assignments, **C.)** PCA using genetic cluster assignments, and **D)** DAPC using genetic cluster assignments

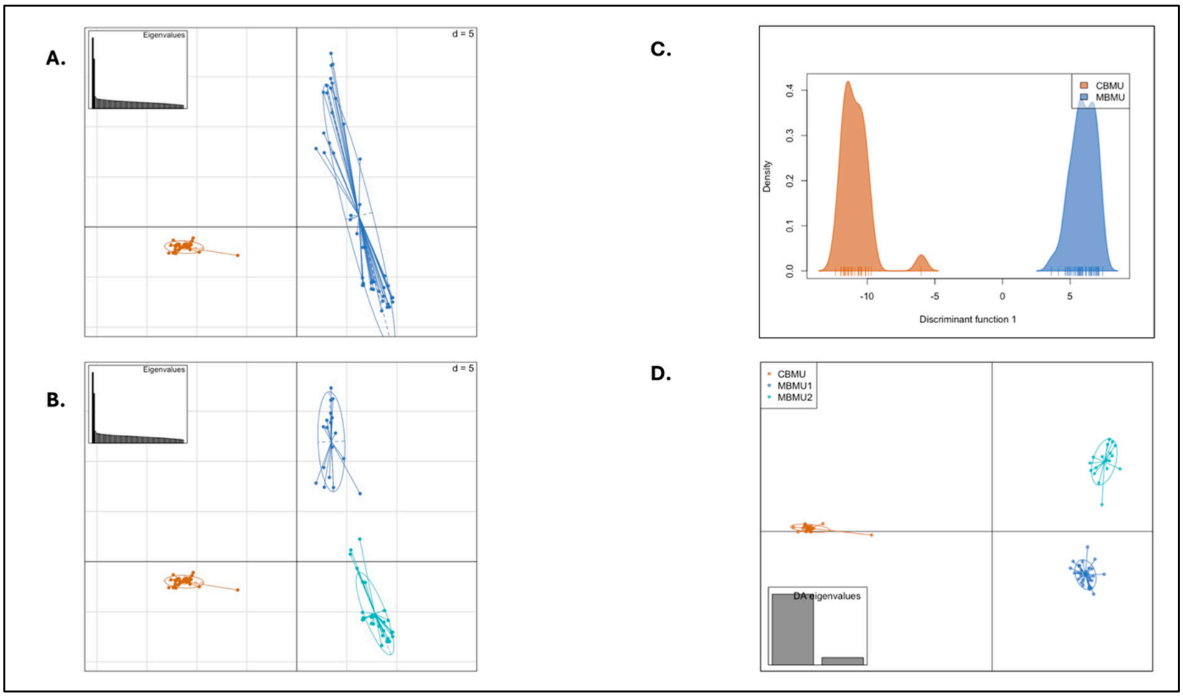

Supplement: Supplementary file 1 [file genes-17-01206-s001.zip › Supplemental_Figures_-_BearManuscript.pdf]
